# Supplementary material for: Cryo-EM structures reveal the H+/citrate symport mechanism of Drosophila INDY
Source: Life Sci Alliance. 2025 Jan 30;8(4):e202402992. doi: 10.26508/lsa.202402992 (PMC11782487; doi:10.26508/lsa.202402992)
Supplement: Supplementary file 4 [file LSA-2024-02992_TableS1.docx]

**Table S1. Cryo-EM data collection, refinement and validation statistics for apo INDY.**

|  | apo | | | |
| --- | --- | --- | --- | --- |
|  | outward-open  (pH 6)  PDB ID 8ZL1  EMD-60215 | asymmetric  (pH 6)  PDB ID 8ZKW  EMD-60210 | inward-open  (pH 8)  PDB ID 8ZL6  EMD-60220 | asymmetric  (pH 8)  PDB ID 8ZKZ  EMD-60213 |
| **Data collection and processing** | | | | |
| Microscope | Titan Krios | | Titan Krios | |
| Camera | K3 BioQuantum | | K3 BioQuantum | |
| Voltage (kV) | 300 | | 300 | |
| Magnification | 105,000 | | 105,000 | |
| Total movies (no.) | 11,663 | | 11,663 | |
| Electron dose (e^-^/Å^2^) | 60 | | 60 | |
| Defocus range (μM) | -1.0 ~ -2.2 | | -0.8 ~ -2.2 | |
| Pixel size (Å) | 0.826 | | 0.851 | |
| Exposure time (s) | 2.9 | | 2.9 | |
| Initial particles (no.) | 1,915,678 | | 3,706,214 | |
| Final particles (no.) | 605,194 | 801,142 | 281,446 | 599,067 |
| Symmetry | C1 | C1 | C2 | C1 |
| B-factor sharpening (Å^2^) | 128.1 | 115.5 | 125.3 | 111.6 |
| Map resolution (Å) | 2.8 | 2.7 | 2.8 | 2.7 |
| **Model refinement** | | | | |
| No. atoms | | | | |
| Protein | 8,366 | 8,376 | 8,380 | 8,376 |
| POPE | 100 | 100 | 100 | 100 |
| N-acetyl-beta-D-glucosamine | - | 28 | 28 | 14 |
| B factors | | | | |
| Protein | 140.6 | 132.5 | 153.9 | 126.4 |
| POPE | 142.1 | 135.7 | 146.6 | 122.8 |
| N-acetyl-beta-D-glucosamine | - | 162.2 | 179.8 | 153.0 |
| RMS deviations | | | | |
| Bond lengths (Å) | 0.003 | 0.005 | 0.005 | 0.007 |
| Bond angles (°) | 0.5 | 0.6 | 0.6 | 0.6 |
| Molprobity score | 1.5 | 1.5 | 1.2 | 1.2 |
| Clash score | 7.9 | 8.0 | 5.3 | 3.3 |
| Ramachandran plot | | | | |
| Favored (%) | 97.3 | 97.4 | 98.2 | 97.3 |
| Allowed (%) | 2.7 | 2.6 | 1.8 | 2.7 |
| Outliers (%) | 0.0 | 0.0 | 0.0 | 0.0 |
