# Supplementary material for: Cryo-EM structures reveal the H+/citrate symport mechanism of Drosophila INDY
Source: Life Sci Alliance. 2025 Jan 30;8(4):e202402992. doi: 10.26508/lsa.202402992 (PMC11782487; doi:10.26508/lsa.202402992)
Supplement: Supplementary file 5 [file LSA-2024-02992_TableS2.docx]

**Table S2. Cryo-EM data collection, refinement and validation statistics for citrate (or DIDS)-bound INDY.**

|  | citrate-bound | DIDS-bound | |
| --- | --- | --- | --- |
|  | inward-partially open  (pH 6)  PDB ID 8ZL4  EMD-60218 | outward-open  (pH 6)  PDB ID 8ZL3  EMD-60217 | asymmetric  (pH 6)  PDB ID 8ZL2  EMD-60216 |
| **Data collection and processing** | | | |
| Microscope | Talos Artica | Titan Krios | |
| Camera | K3 BioQuantum | K3 BioQuantum | |
| Voltage (kV) | 200 | 300 | |
| Magnification | 100,000 | 105,000 | |
| Total movies (no.) | 16,820 | 8,431 | |
| Electron dose (e^-^/Å^2^) | 49.9 | 60 | |
| Defocus range (μM) | -1.2 ~ -2.4 | -0.8 ~ -2.0 | |
| Pixel size (Å) | 0.830 | 0.851 | |
| Exposure time (s) | 3.4 | 2.9 | |
| Initial particles (no.) | 12,386,284 | 2,797,705 | |
| Final particles (no.) | 520,040 | 447,662 | 483,241 |
| Symmetry | C1 | C1 | C1 |
| B-factor sharpening (Å^2^) | 185.0 | 113.9 | 113.2 |
| Map resolution (Å) | 3.6 | 2.9 | 2.8 |
| **Model refinement** | | | |
| No. atoms | | | |
| Protein | 8,442 | 8,366 | 8,396 |
| citrate | 26 | - | - |
| DIDS | - | 56 | 28 |
| POPE | 100 | 100 | 100 |
| N-acetyl-beta-D-glucosamine | 28 | 28 | 28 |
| B-factors | | | |
| Protein | 151.7 | 135.6 | 85.5 |
| citrate | 165.7 | - | - |
| DIDS | - | 174.5 | 112.6 |
| POPE | 147.6 | 139.5 | 86.3 |
| N-acetyl-beta-D-glucosamine | 210.8 | 179.6 | 114.9 |
| RMS deviations | | | |
| Bond lengths (Å) | 0.005 | 0.006 | 0.004 |
| Bond angles (°) | 0.7 | 0.6 | 0.5 |
| Molprobity score | 1.4 | 1.1 | 1.0 |
| Clash score | 8.7 | 3.2 | 2.7 |
| Ramachandran plot | | | |
| Favored (%) | 98.1 | 98.2 | 98.5 |
| Allowed (%) | 1.9 | 1.8 | 1.5 |
| Outliers (%) | 0.0 | 0.0 | 0.0 |
